# Testosterone Therapy in Women: A Literature Review of Clinical Indications, Dosing, Routes of Administration, and Safety

**DOI:** 10.7759/cureus.106713

**Published:** 2026-04-09

**Authors:** Juana Montero Bernaldez, Elizabeth R Burgess, Rebeca Martinez, Manuel Peñalver

**Affiliations:** 1 Obstetrics and Gynecology, Florida International University, Herbert Wertheim College of Medicine, Miami, USA

**Keywords:** female sexual dysfunction, hormone replacement therapy, hypoactive sexual desire disorder (hsdd), menopause, testosterone therapy

## Abstract

Testosterone (T) therapy has been used to address symptoms in women such as low libido, fatigue, and mood disturbances. Despite nearly a century of global use and consistent patient demand, the Food and Drug Administration (FDA) has yet to approve a T formulation for women. This narrative review examines the physiological role of T in females, the clinical presentation of androgen insufficiency, and the evidence supporting T therapy in both pre-and post-menopausal women.

Androgens are critical to female health, with T being the most abundant biologically active sex androgen in women. It is produced in the ovaries and adrenal glands and acts via androgen receptors found throughout the body. T levels in women decline gradually with age and more sharply following menopause or surgical oophorectomy, often resulting in symptoms like hypoactive sexual desire disorder (HSDD), vasomotor symptoms, and mood changes. Diagnostic challenges persist due to inconsistent laboratory assays and hormonal variability, but a symptom-based clinical approach is increasingly recognized as more reliable than relying solely on serum levels.

Evidence from decades of clinical use and numerous studies supports the safety and effectiveness of T therapy, particularly via subcutaneous implants and transdermal patches. Although concerns exist around supraphysiologic doses, these are often necessary for symptom relief and are generally well-tolerated. Adverse effects are typically mild and reversible, with acne and hirsutism being the most common. Existing long-term safety data, including from transgender medicines, have not yet shown increased risks of breast cancer or cardiovascular disease at therapeutic doses.

In the absence of FDA-approved formulations for women, many patients rely on compounded therapies with variable quality and oversight. This regulatory gap limits access to standardized, evidence-based care. Given the widespread and growing use of T in women, long-term randomized controlled trials (RCTs) are urgently needed to establish clear dosing guidelines, safety parameters, and approval pathways. Women deserve access to regulated, effective treatments grounded in scientific evidence.

## Introduction and background

Subcutaneous testosterone (T) therapy delivered by implants to relieve perimenopausal and postmenopausal symptoms in females has been practiced from 1930 to the present day. All over the world, millions of females have used this therapy with no serious side effects reported [1-6]. South Africa and Australia have already approved its use for more than 60 years [6]. Today, its use is growing largely due to patient demand driven by the failure of estrogen (E) therapy to improve all menopausal symptoms, particularly sexually related ones. Although the Food and Drug Administration (FDA) has approved hormone-replacement therapy that is E-based, it has yet to approve the use of T therapy for use in women in America.

In 2004, the FDA denied approval of a 300-µg T patch for the treatment of hypoactive sexual desire disorder (HSDD), which showed reassuring safety profiles over two years, citing insufficient long-term safety data and uncertainty regarding efficacy, leaving E therapy as the only approved option [6,7]. Although the need for extended safety data is valid, the absence of FDA-approved options persists despite decades of widespread T use among American women without clear evidence of significant harm. Additionally, many menopausal symptoms are not directly attributable to estradiol deficiency [8], highlighting the need for alternative therapies. Accordingly, we urge the FDA to support a rigorous, long-term randomized controlled trial (RCT) to definitively evaluate the safety and efficacy of T therapy in this population.

This article provides a narrative review of T therapy in females, including symptoms, side effects, dosing strategies, and routes of administration. A PubMed search was conducted using the terms “testosterone” and “female” to identify relevant studies, reviews, and clinical reports. As a narrative review, this work synthesizes existing evidence without employing a systematic review protocol or formal PRISMA methodology. The goal is to encourage the FDA to initiate long-term RCTs and begin the approval process for a therapy that has demonstrated extensive historical safety and patient benefit.

## Review

Physiological role of androgens in women

In 1941, it was established that female bodies synthesize androgens, which are essential precursors for E synthesis [9,10]. Despite this, the common association of androgens with men has limited the understanding of their true significance for women. For example, Luu-The et al. demonstrated that T is the most abundant biologically active sex androgen in women, and Panay and Fenton found that young ovaries produce three to four times more T than E [11,12].

In both males and females, T, androstenedione, dehydroepiandrosterone (DHEA), and dehydroepiandrosterone sulfate (DHEAS) are steroids produced by the gonads and adrenal glands. These are collectively called androgens, which also include the most potent form, dihydrotestosterone (DHT) [13]. In females, approximately 25% of androgen synthesis occurs in the adrenal glands, 23% in the ovaries, and the remaining production occurs in other tissues [11,14]. The total concentration of T in the blood reflects the overall production of androgens [13].

Androgen receptors (AR) are found in many tissues, including the heart, blood vessels, gastrointestinal tract, lungs, bladder, uterus, ovaries, vagina, bone marrow, synovium, breast, pilosebaceous units, skeletal muscle, adipose tissue, and the central nervous system (such as the hypothalamus and limbic system) [15-17]. T acts directly by converting to DHT on these receptors to improve T-related symptoms and also via aromatization of T to estradiol in E-dependent tissues (brain, bone, fat, muscle, cardiac, vascular, and breast tissues). The aromatization can explain why T alone is an effective therapy in postmenopausal females, being able to improve the most common postmenopausal symptoms [8]. However, the fact that T has also been effective when it is used in combination with an aromatase inhibitor suggests that the effects of T are mainly via AR [18,19].

In summary, many studies since 1945 have supported that there is a role for androgens in female physiology. Despite this, the role of androgens in women has been largely ignored, especially when compared to the more than 30 approved T therapies available for males.

Androgen insufficiency etiologies

The classical documented causes of androgen insufficiency (AI) include dysfunction of the hypothalamus or pituitary gland, adrenal insufficiency (e.g., Addison’s disease), ovarian failure, elevated sex hormone-binding globulin (SHBG) levels (as seen with the use of oral contraceptives or oral Es), and theoretical AR resistance. However, because T levels naturally decline with age, aging itself is considered the most common cause of AI.

By the age of 40 years, a woman’s mean T level is roughly half that of a 21-year-old [20,21]. Unlike E, T levels show minimal variation during the natural menopausal transition [13,21,22]. One study found no significant differences in serum T between pre- and postmenopausal women of the same age and also demonstrated that postmenopausal ovaries continue to produce T [21].

Production of DHEA and androstenedione also decreases with age. Although their serum levels are higher than those of T, their decline contributes to a substantial drop in bioavailable T at ARs [21,23-26]. DHEA levels can be thousands of times higher than T, and androstenedione 5-10 times higher.

Other factors to consider include ARs and SHBG. With aging, AR may become resistant to T, similar to how insulin resistance develops [27]. This theory is difficult to confirm, however, as the molecular structure of the AR is still not fully understood [28].

In summary, T levels decline with age, with the steepest drop occurring during the reproductive years [29]. This helps explain why many premenopausal women report “menopausal symptoms” that are not primarily E-related [30].

Clinical symptoms and diagnosis

The most frequent symptoms of AI include changes in sexual function (such as decreased libido), persistent and unexplained fatigue, dysphoric mood (including depression, irritability, and anxiety), and vasomotor symptoms (e.g., hot flashes) [13]. Additionally, HSDD may occur. HSDD is characterized by distress due to a loss or reduction of sexual interest, affecting 1 in 10 women. Its prevalence is higher in young women with surgical menopause (16-26%) than in naturally premenopausal women (7-14%) [21,31]. These findings support the understanding that declining androgens with age can lead to HSDD, even before menopause [8].

In 2001, a panel of experts convened in Princeton, New Jersey, to evaluate existing evidence on low androgen levels and sexual dysfunction in women. The meeting resulted in the first formal definition of AI as a clinical syndrome, marking a significant step in acknowledging the relationship between androgen levels and sexual symptoms in both pre- and postmenopausal women. This new syndrome was defined based on the following three criteria: (1) presence of common AI symptoms (decreased libido, persistent fatigue, reduced well-being or mood disturbances, and vasomotor symptoms such as hot flashes) [13], (2) adequate E levels (either via natural menstrual cycles in premenopausal women or E therapy in postmenopausal women), and (3) T levels at or below the lowest quartile of the normal range for women aged 20-40 years.

However, limiting this diagnosis to women with adequate E contradicts evidence showing that androgens, especially T, are essential to female sexual function [8,17,28,32-35]. T plays a key role in maintaining libido and sexual desire in women [36], and there is limited evidence linking E levels to sexual function, particularly sexual desire [29,37]. In fact, T alone has been shown to improve many postmenopausal symptoms through both direct effects and aromatization to E [8,30,38].

Several points should be considered regarding the third diagnostic criterion. T values fluctuate due to numerous biological and lifestyle factors, including circadian rhythms, menstrual cycles, seasonal variations (higher in autumn, lower in summer), diet (e.g., SHBG decreases with high-protein, high-fat intake), physical activity (lower in athletes), illness, sexual activity (increases T), smoking (increases T), stress (decreases T), and ethnicity [23,39-49]. In addition, significant analytical variability exists even within the same laboratory, depending on sample handling, storage, and testing methods [43,50,51].

These challenges are especially pronounced at the low T levels typical in women, which decline further with age and menopause [52-55]. Moreover, the tissue-specific conversion of androgen precursors (such as DHEA and androstenedione) to T is not measurable at the cellular level [26]. Due to the limited accuracy and precision of many clinical assays for total and free T in women, there has been growing support for standardizing testing methodologies [56]. Although liquid or gas chromatography with tandem mass spectrometry offers superior accuracy and reproducibility, it remains costly and technically complex [57].

These methodological limitations may explain why several studies found no correlation between baseline levels of estradiol or free T and symptom severity [8,27]. Similar findings from other research highlight the lack of a consistent relationship between T levels and AI-related symptoms [7,8,58].

In summary, defining a “normal” T range in women is extremely difficult [23,59]. These challenges stress the importance of prioritizing clinical evaluation, using a scale such as the Menopause Rating Scale (MRS), over serum values in diagnosing AI [60].

Treatment

Before initiating T therapy for women with HSDD, it is essential to rule out other contributing factors such as relationship issues, concurrent medications, depression or mood disorders, genitourinary syndrome of menopause (GSM), and other potential causes such as anemia [61]. Additionally, HSDD should be distinguished from female sexual arousal disorder (FSAD), as the two conditions have different etiologies and treatment approaches [6]. Although serum T levels do not correlate with the presence or absence of HSDD or its severity, there is a correlation between T concentration during therapy and improvement in sexual desire [62]. 

Concerns about the true efficacy of T in improving sexual function were among the reasons the FDA declined approval of T therapy for women in 2004, leaving E as the only approved hormone therapy. However, the use of androgens in women dates back to 1941, initially for conditions such as fibroids, breast cancer, and low libido. Even then, a sense of improved well-being was noted in female patients [63-65]. Since that time, T therapy has changed formulations and delivery systems over the years and has been used successfully to treat HSDD, with numerous studies supporting its effectiveness [66-69]. Between 1990 and 2000 alone, over 100 articles were published on the topic [70].

In 2010, Glaser et al. reported statistically significant improvements in psychological, somatic, and urogenital symptoms using validated health-related quality of life (HRQOL) and menopausal rating scales in both premenopausal and postmenopausal women treated with subcutaneous T pellets [8]. More recently, a 2019 meta-analysis by Islam et al., which included 36 RCTs and 8,480 participants, demonstrated that transdermal T therapy significantly improved HSDD symptoms in women [71].

Dosage

A central controversy in T therapy for women involves whether to maintain serum T levels at “physiological” concentrations, comparable to endogenous levels in premenopausal women, or allow “pharmacological” (supraphysiological) levels [60]. Higher serum T concentrations are more often achieved with subcutaneous pellets and intramuscular injections, whereas transdermal formulations typically maintain levels within the physiological range [1,26,72-74].

However, studies have shown that theoretical physiological doses may be clinically insufficient, with supraphysiological levels required for meaningful symptom relief [8,26,72,74-76]. Glaser et al. validated this using the MRS, finding that higher serum T levels were associated with greater improvements in quality of life [8]. These findings align with other research suggesting that most benefits of T, except for improvements in vaginal dryness and anxiety, are dose-dependent [2,8,66,73,76-79]. Additionally, Glaser et al. observed that heavier women required higher doses for symptom resolution, supporting the concept of weight-based dosing [3,8].

In practice, subcutaneous T doses ranging from 55 to 240 mg, used since 1938, have been shown to be effective, well-tolerated, and safe [1,2,4,5,8,19,26,72,73,78-81]. An average starting dose of 2 mg/kg with T implants has also demonstrated both safety and clinical efficacy [8,72,73,80].

Conversely, several studies have found that transdermal T therapy, which maintains physiological T concentrations, is effective in treating HSDD in postmenopausal women, whether natural or surgical [57]. A 300 µg/day transdermal patch has shown significant benefit in multiple double-blind, placebo-controlled RCTs [81-84]. Islam et al.’s 2019 meta-analysis of 36 RCTs (8,480 patients) similarly found that transdermal T improved HSDD symptoms and had a more favorable lipid profile compared to oral formulations [71].

Despite this, the 2019 Global Consensus Position Statement on Testosterone Therapy for Women recommended against any T preparations that produce supraphysiological serum concentrations, such as injections and pellets [57]. However, it is important to note that serum T levels are difficult to measure accurately, and there is no established cutoff to distinguish women with or without sexual dysfunction [57]. Multiple studies confirm that neither symptoms of AI nor response to therapy reliably correlate with serum T levels [77,85,86].

In summary, AI remains a clinical diagnosis. Treatment decisions and dose adjustments should be based on symptomatology and therapeutic response rather than laboratory thresholds alone [58].

Routes of administration

T can be administered via several routes, including oral, intramuscular, subcutaneous, and transdermal (patch or topical). Oral T has been associated with unfavorable lipid profile changes, specifically increasing LDL and decreasing HDL cholesterol. Intramuscular T, although effective, is primarily used in transgender men and in the treatment of male hypogonadism [86].

Subcutaneous

Subcutaneous T has been administered in pellet form since 1936. These implants, typically inserted under the skin in the abdominal or gluteal region, provide a sustained release of T over several months. First described in 1950, T pellets were favored for mimicking the body's natural hormone secretion, with one early report stating they “more nearly approached the endogenous mechanism of hormone secretion in the normal female organism” [87].

Glaser et al.'s observational study (2012) further supported this by measuring serum T every two hours over a 26-hour period for six weeks following pellet insertion, demonstrating circadian fluctuations that mirror the body’s natural androgen production [26].

Unlike oral T, the subcutaneous route bypasses hepatic metabolism, thereby avoiding effects on clotting factors and reducing thrombotic risk [88,89]. It also tends to have neutral or even beneficial effects on lipid profiles, with some studies reporting increases in HDL and reductions in VLDL and triglycerides [1,72,80,88]. In premenopausal women, T pellets do not disrupt the menstrual cycle; however, contraception is still necessary to prevent pregnancy during treatment [90-92]. Additionally, the pellets fully dissolve over time and do not require removal.

The safety, efficacy, and circadian-aligned hormone delivery of subcutaneous pellets are well-supported in peer-reviewed literature [8]. Potential benefits include improvement in menopausal symptoms, HSDD, quality of life, and possibly favorable effects on body composition (muscle mass) and metabolic markers [93]. Still, concerns exist about their availability, as these pellets are only accessible through compounding pharmacies. Their use is considered appropriate when such pharmacies comply with quality and safety standards, including those for active pharmaceutical ingredients (APIs) and good manufacturing practice (GMP) [57].

Transdermal

RCTs using 300 µg T patches, considered as physiological doses, in postmenopausal women have shown reassuring safety profiles [91]. Importantly, transdermal T has not been associated with changes in liver function, blood counts, lipid profiles, coagulation parameters, or carbohydrate metabolism [57,84,94,95]. The most commonly reported adverse events are mild skin reactions at the patch application site [94]. Unlike subcutaneous T implants, the transdermal route is non-invasive and can be easily discontinued if adverse effects arise. However, current clinical trial data are insufficient to recommend one specific method of androgen replacement over another [13].

Safety

RCTs evaluating T therapy in women have not exceeded two years in duration, limiting long-term data on outcomes such as cardiovascular health, breast, and endometrial cancer risk, and cognitive effects in both pre- and postmenopausal women [61]. Despite this, T therapy has been used in women for over 90 years without reports of serious adverse events [1].

Existing evidence supports the safety and efficacy of T implants, even at supraphysiological doses [1-5]. Long-term use in transgender men (female-to-male therapy), excluding effects from aromatization, has not identified a maximum dose associated with serious adverse outcomes [81,96,97].

The most commonly reported side effects are acne and hirsutism, both of which are dose-dependent and typically reversible with dose reduction [30,38]. More significant virilizing effects, such as clitoral enlargement or voice deepening, are rare and usually occur at supraphysiological doses; when present, these may be irreversible [84]. Alopecia does not appear to be a consistent concern. In fact, women with pre-existing hair thinning often report regrowth during subcutaneous T therapy, a finding supported by studies showing increased scalp hair and no evidence of androgenetic alopecia at physiological doses [57,72].

As previously mentioned, safety concerns persist regarding compounded T formulations. The 2019 Global Consensus Position Statement supports the reasonable use of compounded products provided they (1) meet rigorous standards for ingredient quality and manufacturing practices and (2) that no approved female T products are available [57]. In the absence of approved female T formulations, it is considered reasonable to use male T preparations as long as serum concentrations remain within the female physiological range.

Breast Cancer

The relationship between T therapy and breast cancer risk remains controversial. Some studies suggest an increased risk, whereas others report a decreased risk, citing the antiproliferative effects of T on breast tissue [98-100,101,102]. Several investigations have even demonstrated a protective effect with supraphysiological doses of T [5,100-104]. Notably, T has been used safely in women with breast cancer at high doses (500-1800 mg) via subcutaneous implants [76]. However, because women with a history of breast cancer have been excluded from RCTs evaluating T for HSDD, its use in this population is generally discouraged [57].

Importantly, T therapy does not appear to increase mammographic breast density [57]. Short-term RCTs and systematic reviews have shown that physiological doses of transdermal T do not elevate breast cancer risk [57,100,105]. For example, a 52-week trial of 279 menopausal women using transdermal T without concurrent E therapy reported only minimal changes in breast density, with no significant difference between T and placebo groups [71].

In transgender therapy (female to male), long-term use of supraphysiological doses has also been found to be safe, with no increased incidence of breast cancer reported [76,81,96,97]. Additionally, subcutaneous T implants are not excreted into breast milk and are considered safe during breastfeeding [75]. However, due to the lack of long-term, large-scale studies specifically in women, the true risk of breast cancer associated with T therapy remains difficult to definitively determine [106,107,108].

Endometrial Cancer

T therapy alone does not require endometrial protection [109,110].

Cardiovascular Disease

Men are known to have a higher risk of cardiovascular disease than women. However, women with elevated androgen levels, such as those with polycystic ovary syndrome (PCOS), also show increased cardiovascular risk, raising concerns that androgens may be atherogenic in females [111]. Oral T is not recommended due to its adverse impact on lipid profiles [57].

However, a short-term RCT (12-24 months) using physiological doses of transdermal or injectable T (equivalent to 300 µg) in premenopausal women did not show harmful effects on lipid profiles, cardiovascular disease, blood pressure, blood glucose, or HbA1c [57]. The 2019 Global Consensus Position Statement supports these findings, stating that T therapy in women is not associated with increases in blood pressure, glycemic markers, or thrombotic risk [57].

One 16-month RCT using transdermal T in older women with heart failure demonstrated improvements in muscle strength, insulin resistance, and functional capacity, although these results have yet to be replicated in larger trials [112]. T implants at certain doses can be relatively safe and may confer cardiovascular benefits [92,113]. Additionally, T therapy has not been associated with an increased risk of deep vein thrombosis [57].

It is important to note, however, that most RCTs have excluded women with high cardiometabolic risk. As a result, data on the safety of T therapy in this population remain limited, and caution is advised when considering treatment in women with elevated cardiovascular risk [57].

## Conclusions

HSDD is a prevalent and clinically significant condition in women. There is substantial evidence supporting T therapy as an effective treatment for HSDD, and it is currently the only evidence-based indication for T use in women. Despite this, no FDA-approved T formulations are currently available for female patients, in contrast to the numerous options approved for men. This disparity stems largely from ongoing safety concerns. It remains notable that, despite decades of clinical use and extensive patient exposure, no FDA-approved T formulation currently exists for women. This is especially striking given the growing demand, largely driven by the failure of E therapy to adequately address sexual symptoms.

This regulatory gap has created space for the widespread use of unregulated and potentially unsafe T preparations, and in some cases, for non-medical, cosmetic use. Women deserve access to safe, standardized, evidence-based treatments. To move toward this goal, the FDA should prioritize (1) initiating long-term RCTs that specifically evaluate breast cancer risk and cardiovascular health, (2) establishing standardized dosing guidelines that account for both physiological and supraphysiological symptom relief, and (3) approving regulated formulations designed for women rather than relying on compounded products or off-label male formulations. These steps would directly address the major barriers identified in this review and lay the foundation for safe, effective, and equitable care. We urge the FDA to address this long-standing oversight and prioritize research and regulatory action for T therapy in women.
